# Detailed estimation of bioinformatics prediction reliability through the Fragmented Prediction Performance Plots

**DOI:** 10.1186/1471-2105-8-380

**Published:** 2007-10-11

**Authors:** Oliviero Carugo

**Affiliations:** 1 Department of General Chemistry, Pavia University, Viale Taramelli 12, I-27100 Pavia, Italy, and Department of Biomolecular Structural Chemistry, Max F. Perutz Laboratories, Vienna University, Campus Vienna Biocenter 5, A-1030 Vienna, Austria

## Abstract

**Background:**

An important and yet rather neglected question related to bioinformatics predictions is the estimation of the amount of data that is needed to allow reliable predictions. Bioinformatics predictions are usually validated through a series of figures of merit, like for example sensitivity and precision, and little attention is paid to the fact that their performance may depend on the amount of data used to make the predictions themselves.

**Results:**

Here I describe a tool, named Fragmented Prediction Performance Plot (FPPP), which monitors the relationship between the prediction reliability and the amount of information underling the prediction themselves. Three examples of FPPPs are presented to illustrate their principal features. In one example, the reliability becomes independent, over a certain threshold, of the amount of data used to predict protein features and the intrinsic reliability of the predictor can be estimated. In the other two cases, on the contrary, the reliability strongly depends on the amount of data used to make the predictions and, thus, the intrinsic reliability of the two predictors cannot be determined. Only in the first example it is thus possible to fully quantify the prediction performance.

**Conclusion:**

It is thus highly advisable to use FPPPs to determine the performance of any new bioinformatics prediction protocol, in order to fully quantify its prediction power and to allow comparisons between two or more predictors based on different types of data.

## Background

Bioinformatics prediction methods rely systematically on the knowledge stored in biological databases and consequently the prediction reliability depends on the amount and quality of the available information.

For example, early predictions of protein secondary structure based on amino acidic sequence were rather unreliable, since the paucity of protein three-dimensional structures that were available [1]. Later on, both the growth of the knowledge embedded in the Protein Data Bank [2,3] and the use of evolutionary information, taken from protein sequence databases and examined with multiple sequence alignments, made secondary structure predictions much more reliable [4,5]. Presently, several methods for predicting the local backbone conformation of residues in proteins can be used as routine tools in molecular biology [6-8].

On the one hand it is obvious that better predictions are possible if larger amounts of experimental information are available: *ab absurdo*, without knowledge, no predictions can be made. However, on the other hand, it is impossible to foresee that a prediction method will become infallible if an infinite number of experimental data become available. This uncertainty can also be seen from a different perspective: It is in general rather difficult to compare the reliability of two (or more) prediction methods [9]. In fact, besides technical difficulties, due to the fact that some of them might not be available as publicly accessible computer programs, they should be compared on the same data sets, something that it is not always possible, given the dynamic nature of biological databases, where new entries may substitute old entries (the Protein Data Bank is a typical example), and also given that usual database browsing tools were not created for this reason. As a consequence, for example, it is often impossible to extract exactly the same set of proteins, characterized by a certain feature, like for example the sub-cellular location or the dimension, by scanning the same database at different dates.

In the present paper, I describe a new procedure to estimate if the reliability of a prediction algorithm is expected to vary with the amount of experimental information that is being accumulated. In other words, this is a way to verify if the quality of the prediction results can be considered to be independent of the amount of experimental information that underlies them, provided that sufficient information is available. If this is verified, it is possible to assume that the prediction method reached its best performance and thus it is also possible to compare two (or more) prediction algorithms independently of the data that were used to develop and validate them.

## Results

### The concept of Fragmented Prediction Performance Plots

In a simple case in which it is necessary to predict if a protein is associated with feature A or with feature B (A and B can be two alternative, mutually exclusive features, like for example two types of secondary structure, two sub-cellular locations, etc.), it is necessary to build two learning sets, one containing type A proteins and the other containing type B proteins. A query protein can then be predicted to be associated with feature A or with feature B by comparing it with the two learning sets [10]. From an algorithmic perspective, such a procedure has an astronomical number of variations, depending on which type of variables are used to represent each subject and on which measure of proximity is used to compare the query protein with the learning sets. The quality of the final predictions is then estimated by verifying if the feature of the query is predicted correctly, by using several queries, the features of which are experimentally known.

The reliability of the predictions is usually estimated by a Jack-knife procedure, in which each member of the learning sets is used as query. It is thus eliminated from the learning sets used to delineate the prediction method and if both learning sets contain N members, such a procedure is performed 2N times and the resulting estimation of the quality of the predictions is based on 2N queries. Obviously, if N is small, one is expecting bad quality predictions. However, little attention is usually put on the eventuality that N is sufficiently large to allow to make reasonable predictions.

Alternatively, several machine learning methods often adopt a partial cross-correlation validation. The learning sets are divided into n subsets, and one of them is used to compare the reality with the predictions performed by using all the others n-1 [11]. This is particularly convenient when a complete Jack-knife test would be computationally too expensive, though it is a simple variants of it. Also in these cases, however, the relationships between learning set dimension and prediction reliability is usually neglected.

To solve such a problem, it is possible to select M elements from both learning sets, with M<<N, and to define and test the prediction method on two subsets of the learning sets, each containing M elements. The reliability R(M) can be thus estimated. Subsequently, it is possible to repeat everything by using two learning sets containing 2M elements each and record the reliability R(2M), and so on until M = N. This results into a series of reliability values R(X) (with X = M, 2M, 3M, etc.) that can be plotted against X. This is defined here as the Fragmented Prediction Performance Plot (FPPP) and some of its properties can be evidenced here.

At low X values, when the learning sets are small, the reliability of the predictions cannot be estimated well. It may be either very small or very large but this does not provide, in general, any consistent information. By increasing X, the prediction reliability tends to converge towards a stable value. If X is large enough, the reliability should be invariant relative to a further increase of X. Such a reliability value can be considered to be the intrinsic reliability of the prediction method.

However, if the number N of proteins contained in the two learning sets is not large enough, such a plateau is not observed in the Fragmented Prediction Performance Plot. In such a case, one can foresee that the reliability of the prediction method will vary if new data will be inserted into the learning sets. In other words, given that the experimental information stored in databases increases quite rapidly, the prediction method should be re-tested at a later date, when new data will become available.

Interestingly, if it is possible to determine the intrinsic reliability of two prediction protocols, it is also possible to compare their performances, independently of the fact that they had been developed by using different learning sets. This is particularly interesting, since, as mentioned in the introduction, it is often rather difficult if not impossible to reconstruct data sets used by different scientists at different dates. Furthermore, the intrinsic reliability values allow one to compare the performances of methods that are based on different types of data, like for example amino acidic sequences and three-dimensional structures, where identical learning sets cannot be assembled by definition.

In the following sections, three prediction methods are described, together with their Fragmented Prediction Performance Plots. They are not intended to make predictions suitable to solve real biological and biophysical problems. They are only examples, selected amongst many others, for describing some properties of the FPPPs.

### General considerations about the predictors

All the examples below share some common features. There are always two learning sets, each containing a particular type of subjects. The predictions are made by comparing the query with each of the two sets and by assigning the query to its closest group. In each case, a Jack-knife complete cross validation was performed.

However, it is important to observe that the FPPPs can be used also in a general case in which there are more than two learning sets, independently of the definition of proximity between pairs of single subjects or of groups of subjects, and independently of the variables used to represent the subjects.

The degree of reliability of each prediction was estimated with the confusion matrix and some of the figures of merit associated with it. First, by defining arbitrarily which of the two features is positive or negative, the following four quantities were defined: true positives (tp) = number of positive events that are correctly predicted; true negatives (tn) = number of negative event that are correctly predicted; false positives (fp) = number of negative events that are (incorrectly) predicted to be positive; and false negative (fn) = number of subjects that are predicted to be negative despite they are positive.

These four elements of the confusion matrix can be used in a wide variety of ways to summarize by means of a single figure of merit the degree of reliability of a prediction. The following three figures of merits were considered here, since they are widely used.

The sensitivity (known also as recall) was defined as

sensitivity=tptp+fn
 MathType@MTEF@5@5@+=feaafiart1ev1aaatCvAUfKttLearuWrP9MDH5MBPbIqV92AaeXatLxBI9gBaebbnrfifHhDYfgasaacH8akY=wiFfYdH8Gipec8Eeeu0xXdbba9frFj0=OqFfea0dXdd9vqai=hGuQ8kuc9pgc9s8qqaq=dirpe0xb9q8qiLsFr0=vr0=vr0dc8meaabaqaciaacaGaaeqabaqabeGadaaakeaacqqGZbWCcqqGLbqzcqqGUbGBcqqGZbWCcqqGPbqAcqqG0baDcqqGPbqAcqqG2bGDcqqGPbqAcqqG0baDcqqG5bqEcqGH9aqpdaWcaaqaaiabdsha0jabdchaWbqaaiabdsha0jabdchaWjabgUcaRiabdAgaMjabd6gaUbaaaaa@4675@

the precision (sometime referred to also as specificity) was defined as

precision=tptp+fp
 MathType@MTEF@5@5@+=feaafiart1ev1aaatCvAUfKttLearuWrP9MDH5MBPbIqV92AaeXatLxBI9gBaebbnrfifHhDYfgasaacH8akY=wiFfYdH8Gipec8Eeeu0xXdbba9frFj0=OqFfea0dXdd9vqai=hGuQ8kuc9pgc9s8qqaq=dirpe0xb9q8qiLsFr0=vr0=vr0dc8meaabaqaciaacaGaaeqabaqabeGadaaakeaacqqGWbaCcqqGYbGCcqqGLbqzcqqGJbWycqqGPbqAcqqGZbWCcqqGPbqAcqqGVbWBcqqGUbGBcqGH9aqpdaWcaaqaaiabdsha0jabdchaWbqaaiabdsha0jabdchaWjabgUcaRiabdAgaMjabdchaWbaaaaa@436D@

and the Matthews correlation coefficient was defined as

Matthews correlation coefficient=(tp⋅tn)−(fp⋅fn)(tp+fp)(tp+tn)(tn+fp)(tn+fn)
 MathType@MTEF@5@5@+=feaafiart1ev1aaatCvAUfKttLearuWrP9MDH5MBPbIqV92AaeXatLxBI9gBaebbnrfifHhDYfgasaacH8akY=wiFfYdH8Gipec8Eeeu0xXdbba9frFj0=OqFfea0dXdd9vqai=hGuQ8kuc9pgc9s8qqaq=dirpe0xb9q8qiLsFr0=vr0=vr0dc8meaabaqaciaacaGaaeqabaqabeGadaaakeaacqqGnbqtcqqGHbqycqqG0baDcqqG0baDcqqGObaAcqqGLbqzcqqG3bWDcqqGZbWCcqqGGaaicqqGJbWycqqGVbWBcqqGYbGCcqqGYbGCcqqGLbqzcqqGSbaBcqqGHbqycqqG0baDcqqGPbqAcqqGVbWBcqqGUbGBcqqGGaaicqqGJbWycqqGVbWBcqqGLbqzcqqGMbGzcqqGMbGzcqqGPbqAcqqGJbWycqqGPbqAcqqGLbqzcqqGUbGBcqqG0baDcqGH9aqpdaWcaaqaamaabmaabaGaemiDaqNaemiCaaNaeyyXICTaemiDaqNaemOBa4gacaGLOaGaayzkaaGaeyOeI0YaaeWaaeaacqWGMbGzcqWGWbaCcqGHflY1cqWGMbGzcqWGUbGBaiaawIcacaGLPaaaaeaadaGcaaqaamaabmaabaGaemiDaqNaemiCaaNaey4kaSIaemOzayMaemiCaahacaGLOaGaayzkaaWaaeWaaeaacqWG0baDcqWGWbaCcqGHRaWkcqWG0baDcqWGUbGBaiaawIcacaGLPaaadaqadaqaaiabdsha0jabd6gaUjabgUcaRiabdAgaMjabdchaWbGaayjkaiaawMcaamaabmaabaGaemiDaqNaemOBa4Maey4kaSIaemOzayMaemOBa4gacaGLOaGaayzkaaaaleqaaaaaaaa@8BEC@

Several other quantities can be used for estimating the prediction reliability and all of them can be used to produce Fragmented Prediction Performance Plots. The three figures of merit that are examined here (sensitivity, precision, and Matthews correlation coefficient) are however nearly indispensable for the following reasons. First, sensitivity and precision tend to be anti-correlated and monitor different aspects of the prediction. The sensitivity indicates the fraction of positive events that are recognized by the predictor and the precision monitors how many spurious subjects are incorrectly considered to be positive. Both of them may range from 0 to +1, the latter value being associated with perfect predictions. Second, the Matthews correlation coefficient, which can vary from -1 to +1, with higher values indicating better predictions, considers both the true positives and the true negatives as successful predictions and is rather unaffected by sampling biases, which may occur when the dimensions of the learning sets are very different.

### First example

A predictor of protein subcellular location was designed. Given a single protein sequence, it predicts if the protein is cytoplasmic or if it is an integral membrane protein. Each protein chain is represented by its amino acidic composition and it is thus associated with twenty variables, each of which is the percentage of observations of one of the twenty natural amino acids. The proximity between two subjects is estimated by the Euclidean distance computed over these twenty variables and the proximity between a single subject and a group of subjects is defined as the minimal value of the distances between the single subject and all the subjects belonging to the group.

The amino acidic sequences of 928 human cytoplasmic protein and of 565 integral membrane proteins were downloaded from the UniProt database [12] by using the Sequence Retrieval System [13]. Predictions were initially performed on two randomly generated subsets, each containing only 10 entries. Subsequently, the dimension of these two learning sets was increased until 510, in steps of 10 residues. Therefore, the second round of predictions was performed by using two learning sets of 20 subjects, the third round with sets of 30 subjects, and so on.

**Figure 1:**
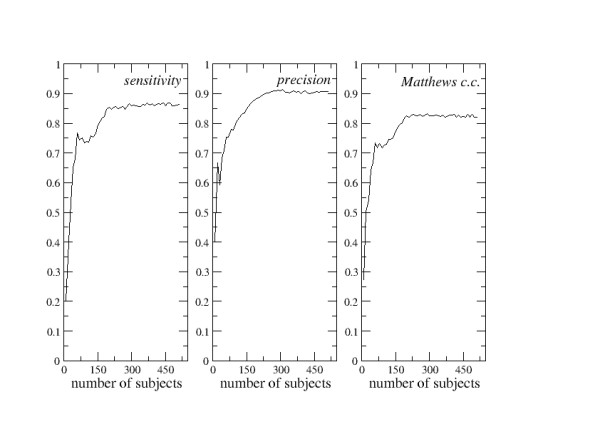
**First FPPP analysis**. Fragmented Prediction Performance Plots for the first example of predictor (see text for details).

The values of sensitivity, precision, and of Matthews correlation coefficient were recorded at each step and are shown in Figure 1.

### Second example

A predictor of quaternary status was designed. It is intended to predict, on the basis of the amino acidic sequence, if a protein chain participates in obligate hetero-oligomeric assemblies with other, different chains or if it exists as a monomeric or a homo-oligomeric protein.

Each chain was represented by a vector of twenty elements, each indicating the percentage of occurrence of one of the twenty natural amino acids within the chain. The proximity between two single subjects **X **= (x1, x2, ..., x20) and **Y **= (y1, y2, ..., y20) was estimated by the Tanimoto coefficient ST, defined as

ST=∑i=120xiyi∑i=120xiyi+∑i=120(xi−yi)2.
 MathType@MTEF@5@5@+=feaafiart1ev1aaatCvAUfKttLearuWrP9MDH5MBPbIqV92AaeXatLxBI9gBaebbnrfifHhDYfgasaacH8akY=wiFfYdH8Gipec8Eeeu0xXdbba9frFj0=OqFfea0dXdd9vqai=hGuQ8kuc9pgc9s8qqaq=dirpe0xb9q8qiLsFr0=vr0=vr0dc8meaabaqaciaacaGaaeqabaqabeGadaaakeaacqqGtbWucqqGubavcqGH9aqpdaWcaaqaamaaqahabaGaemiEaG3aaSbaaSqaaiabdMgaPbqabaGccqWG5bqEdaWgaaWcbaGaemyAaKgabeaaaeaacqWGPbqAcqGH9aqpcqaIXaqmaeaacqaIYaGmcqaIWaama0GaeyyeIuoaaOqaamaaqahabaGaemiEaG3aaSbaaSqaaiabdMgaPbqabaGccqWG5bqEdaWgaaWcbaGaemyAaKgabeaaaeaacqWGPbqAcqGH9aqpcqaIXaqmaeaacqaIYaGmcqaIWaama0GaeyyeIuoakiabgUcaRmaaqahabaGaeiikaGIaemiEaG3aaSbaaSqaaiabdMgaPbqabaGccqGHsislcqWG5bqEdaWgaaWcbaGaemyAaKgabeaaaeaacqWGPbqAcqGH9aqpcqaIXaqmaeaacqaIYaGmcqaIWaama0GaeyyeIuoakiabcMcaPmaaCaaaleqabaGaeGOmaidaaaaakiabc6caUaaa@5E26@

Its values range between -0.33 and +1, if the variables are standardized with

xi=xi∑i=120xi2
 MathType@MTEF@5@5@+=feaafiart1ev1aaatCvAUfKttLearuWrP9MDH5MBPbIqV92AaeXatLxBI9gBaebbnrfifHhDYfgasaacH8akY=wiFfYdH8Gipec8Eeeu0xXdbba9frFj0=OqFfea0dXdd9vqai=hGuQ8kuc9pgc9s8qqaq=dirpe0xb9q8qiLsFr0=vr0=vr0dc8meaabaqaciaacaGaaeqabaqabeGadaaakeaacqWG4baEdaWgaaWcbaGaemyAaKgabeaakiabg2da9maalaaabaGaemiEaG3aaSbaaSqaaiabdMgaPbqabaaakeaadaGcaaqaamaaqahabaGaemiEaG3aa0baaSqaaiabdMgaPbqaaiabikdaYaaaaeaacqWGPbqAcqGH9aqpcqaIXaqmaeaacqaIYaGmcqaIWaama0GaeyyeIuoaaSqabaaaaaaa@3F4C@

and it is a similarity measure between the two subjects that are compared. Therefore, a value equal to +1 indicates the identity between the two subjects and a value equal to -0.33 indicates the complete difference between the two subjects.

The similarity between a single subject and a group of subjects was defined as the maximal value of similarity betweeh the single subject and the entries of the group. A query was considered to participate to a hetero-oligomeric assembly if its similarity to the group of hetero-oligomeric chains was higher that its similarity to the group containing non hetero-oligomeric chains.

The amino acidic sequences of 1406 monomeric protein, 2985 homo-oligomeric proteins, and of 1446 hetero-oligomeric proteins were downloaded from the UniProt database. Initial predictions were performed by using two learning sets, one containing 10 hetero-oligormeric chains and the other containing 10 monomeric and 10 homo-oligomeric protein chains. Subsequently, predictions were performed by enlarging the first learning set to 20 hetero-oligomeric proteins and the other learning set to 20 monomeric and 20 homo-oligomeric proteins, and so on, until the first learning set contained 1400 hetero-oligomeric proteins and the other learning set contained 1400 monomeric and 1400 homo-oligomeric proteins.

**Figure 2:**
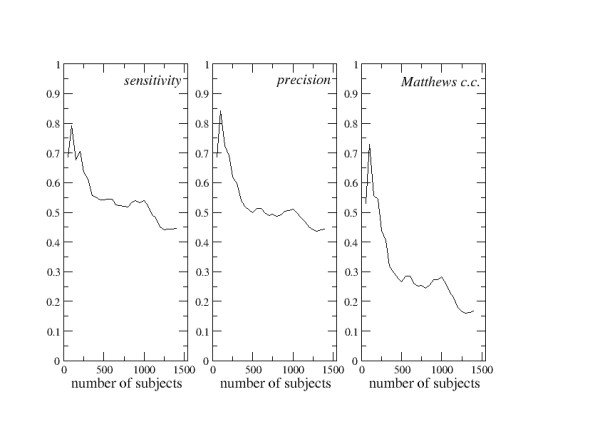
**Second FPPP analysis**. Fragmented Prediction Performance Plots for the second example of predictor (see text for details).

The values of sensitivity, precision, and of Matthews correlation coefficient were recorded at each step and are depicted in Figure 2.

### Third example

This was a variation on the second example described above, the only difference being the definition of proximity between a single subject and a group of subjects. While in the second example the maximal similarity criterion was used, here the similarity between a query chain and a learning set was defined as the average similarity between the single subject and all the entries of the group.

**Figure 3:**
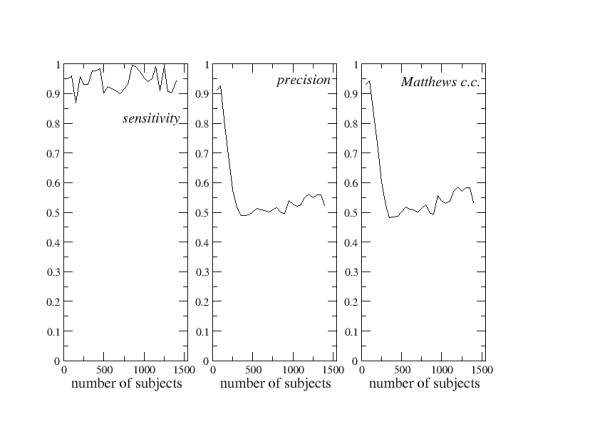
**Third FPPP analysis**. Fragmented Prediction Performance Plots for the third example of predictor (see text for details).

Like in the second example, predictions were performed by enlarging gradually the dimensions of the learning sets and the values of sensitivity, precision, and of the Matthews correlation coefficient were recorded at each step. Figure 3 shows their variation as a function of the learning set dimension.

## Discussion

The Fragmented Prediction Performance Plots describe the variation of the prediction reliability as a function of the amount of information used to make the predictions.

Figure 1 shows the FPPPs obtained in the first example, in which a protein is predicted to be cytoplasmic or membrane-inserted depending on its amino acid composition. If few data are inserted into the learning sets, the values of sensitivity, precision, and of Matthews correlation coefficient are very modest. They rapidly increase if further subjects are included in the learning sets and a plateau is reached at the abscissa value of about 250. For larger learning sets, the values of sensitivity tend to be constant and equal to 0.86. Similarly, the precision tends to converge at 0.91 and the Matthews correlation coefficient at 0.82. These can be considered to be the intrinsic sensitivity, precision, and Matthews correlation coefficient for such a predictor. In other words, this prediction protocol is not expected to provide better results by waiting that new data will accumulate into the databases in the future.

One can observe that these values of sensitivity, precision, and of Matthew correlation coefficient are quite high. And this is not surprising, since it is obvious that soluble, cytoplasmic proteins can easily be distinguished from integral membrane proteins on the basis of their amino acidic composition. It must however be observed that the most reasonable way to further improve the quality of these predictions is not the enlargement of the learning sets. Other variables should be included into the representation of each subject, besides the percentages of the twenty amino acids, and/or other definitions of the proximity between the query and the learning sets should be used. A different algorithmic set-up might also be used.

The FPPPs of the second predictor, which should discriminate protein chains that take part in permanent hetero-oligomeric supra-molecular assemblies from chains that do not, are depicted in Figure 2. It appears that the reliability of such a computational protocol is rater variable if the learning sets are small and tends to decrease by enlarging the learning sets. Even if the Matthews correlation coefficient is still positive, when the learning sets contain 1400 subjects, it can be concluded that this is not a good prediction method.

In fact, not only the values of sensitivity, precision, and of Matthews correlation coefficient are quite modest, but also they tend to decrease when the amount of information stored into the learning sets increases. It is thus reasonable to foresee that when new data will become available in the databases the prediction quality will decrease further. In this example, the intrinsic reliability of the predictions cannot be estimated.

Figure 3 shows the FPPPs of the third predictor, which uses a slightly different algorithmic to solve the same problem encounteres in the second example of predictions. In this case, the sensitivity is very high, close to the maximal value +1, and nearly independent of the dimension of the learning sets. On the contrary both the precision and the Matthews correlation coefficient have the highest value for small learning sets. This is likely to be an unfortunate case. The very few proteins that are used to make the first predictions are casually biased and excellent predictions can be made. When the learning sets enlarge, both the precision and the Matthews correlation coefficient decrease, reach a minimum around the abscissa value of 300, and then increase nearly linearly.

Also in this case it is impossible to define the intrinsic reliability of such a predictor and it can be expected that a further enlargement of the learning sets might allow one to improve the quality of the predictions. In other words, such a prediction protocol seems to be rather promising, though the amount of information included into the data sets seems to be insufficient to fully exploit the potential of this prediction strategy.

The three sets of FPPPs described above (Figures 1, 2, 3) are very different from each other and exemplify most of the information that can be extracted from this type of analysis. In one case (Figure 1), the prediction reliability is rather stable and roughly independent of the dimensions of the learning sets at abscissa values greater than 250. Here, the intrinsic reliability of the predictor can be determined. In the second case (Figure 2), the prediction reliability clearly decreases if the amount of information stored in the learning sets increases. In the third case (Figure 3), the quality of the predictions tends to improve as far as the learning sets are enlarged.

In the experience of the author of the present paper, several good predictors tend to behave like in Figure 1, though many exceptions were observed. In general, it is impossible to foresee which type of trend is presented by a predictor without the analysis of the FPPPs and it seems quite important that the FPPPs of any new bioinformatics prediction tool are determined and analyzed. In most of the cases, this should be rather inexpensive and worthwhile. Actually, it is probably impossible to fully quantify predictor performances without a FPPP analysis.

It must however be observed that the FPPP analysis is not intended to be the only tool to validate the predictors, the performances of which can be influenced by many other factors beside the amount of data that are used to design them. For example, the training sets might contain clusters that behave quite differently and these differences would not diminish even if the data used for training is increased. More in general, three different issues must be considered in order to evaluate predictive technologies: i) it is mandatory to verify if the predictions are better than random guesses; ii) data noise and heterogeneity can seriously affect the prediction quality; iii) different computational methods can behave differently on the same type of input data. On the basis of the three examples described in this paper, it is obvious to observe that the use of larger learning sets cannot per se guarantee an increase of the prediction reliability. However, it is clear that the dependence of the prediction quality on the amount of information present in the learning sets is a very important issue as well, since the final prediction reliability may depend considerably on the learning set dimension. Therefore, although the FPPP analysis, which remembers many bootstrapping procedures, is not the unique tool to validate bioinformatic predictions, it is a useful benchmark along this validation, which is absolutely necessary.

## Methods

All protein sequences were downloaded from the UniProt database [12] by using the Sequence Retrieval System [13] and all computations were written with locally written programs.

## Competing interests

The author declares that there are no competing interests.

## Authors' contributions

OC is the only author of this manuscript.
